# The Association Study of Calmodulin 1 Gene Polymorphisms with Susceptibility to Adolescent Idiopathic Scoliosis

**DOI:** 10.1155/2014/168106

**Published:** 2014-01-16

**Authors:** Yu Zhang, Zuchao Gu, Guixing Qiu

**Affiliations:** ^1^Department of Orthopaedics, Peking Union Medical College Hospital, Beijing 100005, China; ^2^Department of Orthopaedics, The First People's Hospital of Chengdu, Sichuan 610071, China

## Abstract

*Objective.* Idiopathic scoliosis is the most common pediatric spinal deformity affecting 1% to 3% of the population, and adolescent idiopathic scoliosis (AIS) accounts for approximately 80% of these cases; however, the etiology and pathogenesis of AIS are still uncertain. The current study aims to identify the relationship between calmodulin 1 (CALM1) gene and AIS predisposition, to identify the relationship between the genotypes of the SNPs and the clinical phenotypes of AIS. *Methods.* 146 AIS patients and 146 healthy controls were enrolled into this case-control study. 12 single nucleotide polymorphisms (SNPs) candidates in CALM1 gene were selected to determine the relationship between CALM1 gene and AIS predisposition. Case-only study was performed to determine the effects of these variants on the severity of the condition. *Results.* Three SNPs from 12 candidates were found to be associated with AIS predisposition. The ORs were observed as 0.549 (95% CI 0.3519–0.8579, *P* = 0.0079), 0.549 (95% CI 0.3519–0.8579, *P* = 0.0079), and 1.6139 (95% CI 1.0576–2.4634, *P* = 0.0257) for rs2300496, rs2300500, and rs3231718, respectively. There was no statistical difference between main curve, severity, and genotype distributions of all of 12 SNPs. *Conclusion.* Genetic variants of CALM1 gene are associated with AIS susceptibility.

## 1. Introduction

Adolescent idiopathic scoliosis is a structural, tridimensional spinal deformity characterized by lateral curvature of the spine with Cobb angle (which is a measurement used for evaluation of curves in scoliosis [1, 2]) greater than 10°. It affects 2–3% of the adolescent populations [3].

Despite extensive study, the etiology and pathogenesis of AIS are still uncertain [4]; several divergent hypotheses have been postulated to better define this etiology [5, 6]. Genetics, growth hormone secretion, connective tissue structure, muscle structure, vestibular dysfunction, melatonin deficiency, and platelet abnormalities are major areas of research [5, 6]. Adolescent idiopathic scoliosis mainly affects girls in number and severity; several familial surveys of idiopathic scoliosis provided strong evidence that genetic factors have a role in this condition [7]. Single nucleotide polymorphisms (SNPs) in the genes for estrogen receptor a (ESR1) [8], estrogen receptor b (ESR2) [9], matrilin 1 (MATN1) [10], melatonin receptor 1B (MTNR1B) [11], tryptophan hydroxylase 1 (TPH1) [12], interleukin-6 (IL-6), and matrix metalloproteinase-3 (MMP-3) [13] have been reported to be associated with AIS predisposition. However, many scientists believe that there are still some more candidate genes that need to be elucidated.

CALM1 encodes calmodulin (CaM), a ubiquitous eukaryotic calcium-binding protein, which is a principal mediator of the calcium signal [14] and plays an important role in intercellular communication, cell movement, cell differentiation, cell proliferation, and other physiological and biochemical activities [15].

Cantaro et al. [16] found a 2.5- to threefold increase in the level of calmodulin which was an essential mediator of calcium-induced contractility, in the platelets of patients who had idiopathic scoliosis. They also found that the level of calmodulin was associated with the severity of the spinal curve Kindsfater et al. [17] found that platelet CaM in the patient who had a progressive curve (more than 10 degrees/year) was significantly higher than the level in the patients who had a stable curve (less than 10 degrees/year). Lowe et al. [18] also concluded that CaM levels increased in all the patients with progressive curves (13/13), and CaM levels of patients who had a progressive curve (greater than 30° or double structural curves) were higher than the level in the patients who had a stable curve and that of normal control. Therefore, they inferred that the level of platelet CaM appeared to be a useful predictor of progression of the curve than the Risser alone in patients with adolescent idiopathic scoliosis. Inoue et al. [7] found that the XbaI site polymorphism is associated with curve severity; they assumed that it may be correlated with the mechanism of CaM in the curve progression of scoliosis. More importantly, two SNPs, rs12885713 (−16C > T) and rs5871 polymorphisms, in the CALM1 gene have been proved to be predisposing factors for adolescent idiopathic scoliosis [19]. To sum up, CaM is closely related to AIS, but its mechanism is still unclear.

Candidate gene analysis, association analysis, and haplotype analysis are effect methods for investigating complicated multigenetic disease. In order to determine whether polymorphisms of CALM1 are associated with a predisposition to AIS in Chinese Han population, we selected 12 single nuclear polymorphism sites to conduct a case-control study involving 146 AIS patients and 146 control subjects.

## 2. Objects and Methods

### 2.1. Ethics Statement

The study has been approved by the Ethical Committee of the First People's Hospital of Chengdu; written informed consent was obtained from all subjects or their parents in the case of children.

### 2.2. Subjects for the Case-Control Study

A total of 146 patients diagnosed with adolescent idiopathic scoliosis and 146 healthy controls in the First People's Hospital of Chengdu were enrolled in this study between October 2008 and February 2011. All the control subjects were frequency-matched to the cases on age (±3 years), gender, and the Han nationality; curve pattern and Cobb angles of main curve of AIS patients were recorded (Table 1). The following candidates were excluded: those with a history of congenital anomalies, neuromuscular diseases, endocrine disorders, skeletal dysplasia or connective tissue disorders, or mental retardation or mental illness as well as psychiatric patients on medication affecting bone metabolism. From the 146 patients in the scoliosis group, all patients have a Cobb angle above 30 degrees with high risk as they required surgery.

### 2.3. Evaluation of Scoliosis Angle

Normal standing posteroanterior radiographs were taken for each AIS patient upon their first visit. Standard techniques for measuring Cobb's angle were used, and if more than one curve was discovered, the most severe curve was selected for measurement. A Cobb angle of less than 10 degrees was considered normal.

### 2.4. Genotyping Method

Genotypes were screened via single base primer extension assay using an ABI PRISM SNaPshot Multiplex kit (Applied Biosystems, Foster City, CA, USA) according to the manufacturer's recommendations. Briefly, genomic DNA flanking the SNPs of interest was amplified via polymerase chain reaction (PCR) with forward and reverse primer pairs (Table 2) and standard PCR reagents in a 10-microliter reaction volume, containing 10 ng of genomic DNA, 0.5 pM of each oligonucleotide primer, 1 microliter of 10X PCR buffer, 250 M dNTP (2.5 mM each), and 0.25 units of i-StarTaq DNA Polymerase (5 unit/*μ*L) (iNtRON Biotechnology, Seongnam, Korea). The PCR conditions were as follows: 10 min at 95°C for 1 cycle and 35 cycles at 95°C for 30 s, 60°C for 1 min, 72°C for 1 min, followed by 1 cycle of 72°C for 10 min. After amplification, the PCR products were treated with 1 unit each of shrimp alkaline phosphatase (SAP) (USB Corporation, Cleveland, OH, USA) and exonuclease I (USB Corporation, Cleveland, OH, USA) at 37°C for 75 minutes and 72°C for 15 minutes to purify the amplified products. One microliter of the purified amplification products was added to a SNaPshot Multiplex Ready reaction mixture containing 0.15 pmols of genotyping primer for primer extension reaction. The primer extension reaction was carried out for 25 cycles at 96°C for 10 seconds, 50°C for 5 seconds, and 60°C for 30 seconds. The reaction products were then treated with 1 unit of SAP at 37°C for 1 hour and 72°C for 15 minutes to remove excess fluorescent dye terminators. One microliter of the final reaction samples containing the extension products was then added to 9 microliters of Hi-Di formamide (ABI, Foster City, CA, USA). The mixture was incubated at 95°C for 5 min, followed by 5 min on ice, and then analyzed by electrophoresis in an ABI Prism 3730xl DNA analyzer. Analysis was carried out using Gene Mapper software (version 4.0; Applied Biosystems, Foster City, CA, USA). Table 2 shows the primer sets and Tm used for the SNaPshot assay.

### 2.5. Statistical Analysis

Differences between cases and controls with respect to allele frequencies were evaluated using the Chi-Square test using SAS 8.2. All the data of 12 SNPs with polymorphisms were analyzed by the association analysis based on alleles and phenotypes of the SNPs. Odds ratio (OR) and 95% confidence intervals (CI) were computed by the unconditional logistic regression to estimate the relative risk for the single locus genotypes by online software-SNPstats. Haplotype frequencies were estimated and differences in haplotype distributions between cases and controls were assessed by Haploview 4.0 software.

## 3. Results

### 3.1. Epidemiologic Data Analysis

There were no significant differences of age distribution and sex proportion between case group and control group (*P* > 0.05) (Table 1).

### 3.2. Hardy-Weinberg Equilibrium Analysis of the Genotype Distributions between Case Group and Control Group

Hardy-Weinberg equilibrium analysis showed that there was no difference of the genotype distributions of all of 12 SNPs between AIS group and control group (data not shown).

### 3.3. Case-Control Association Analysis Based on SNPs Genotype and Allele

We used the following rules to select SNPs: firstly, we used the software Haploview 4.0 to help us to choose tagSNPs of CALM1; secondly, by NCBI website (http://www.ncbi.nlm.nih.gov/SNP), we preferred to find the SNPs which were located in extron region and 5′ and 3′ untranslated regions; thirdly, we preferred to choose the SNPs whose heterozygosity is above 10% and minor allele frequency (MAF) is above 5%. The distributions of the alleles and genotypes for the 12 SNPs are given in Table 3. Among them, the genotype frequency distributions of rs2300496 (SNP01), rs2300500 (SNP02), and rs3213718 (SNP05) were statistically different between case group and control group (*P* = 0.0196, 0.0196, and 0.0378, resp.). Further analysis revealed that all these three SNPs were all associated with AIS predisposition; the ORs were observed as 0.5495 (95% CI 0.3519–0.8579), 0.5495 (95% CI 0.3519–0.8579), and 1.6139 (95% CI 1.0574–2.4634) for SNP1, SNP2, and SNP5, respectively.

Besides that, distributions of the allele frequency with respect to rs2300496 (SNP01), rs2300500 (SNP02), and rs3213718 (SNP05) were statistically different between case group and control group (*P* = 0.0079, 0.0079, and 0.0257, resp.). Further analysis indicated that C allele of rs2300496, G allele of rs2300500, and C allele of rs3213718 correlated with increased susceptibility to adolescent idiopathic scoliosis (Table 3).

We performed linkage disequilibrium analyses with respect to different SNPs in CALM1 gene using Haploview 4.0, and we found that there was a linkage disequilibrium block in control groups.

As shown in Table 4, haplotype analysis with respect to different SNPs in CALM1 gene using Haploview 4.0 got one positive haplotype: SNP8C-SNP01A-SNPl3C-SNPl4C-SNP02C-SNP3T-SNP4G-SNP05T-SNP011C, its haplotype frequencies were higher than 10%, and that haplotype distributions were significantly different between case group and control group, which again indicated that CALM1 gene was possible to be a susceptibility gene of AIS.

### 3.4. Case-Only Study

#### 3.4.1. Association Analysis between SNPs and Gender in Case Group

Case group was divided into two groups according to gender of patients. Differences with respect to distributions of genotypes for the SNPs were analyzed between these two groups. As we can see from Table 5, none of the SNPs were observed to have significant difference (*P* > 0.05), and no association between the SNPs and gender was detected.

#### 3.4.2. Genotype Polymorphism of the SNPs: Cobb Angles of Scoliosis

In this study, patients of case group included 146 AIS patients with Cobb angles over 30°. We analyzed the association of the SNPs genotype polymorphism with Cobb angles of scoliosis. There was no statistical difference between main curve, severity, and the genotype distributions of all of 12 SNPs.

#### 3.4.3. Association Analysis between SNPs and the Location of Main Curve in AIS

146 patients with AIS were divided into 3 groups including group A (thoracic curve, 90 cases), group B (thoracolumbar curve, 25 cases), and group C (lumbar curve, 31 cases). The curve patterns or characteristics of thoracic curve, thoracolumbar curve, and lumbar curve were that their scoliosis apexes were located in the intervertebral disc space between T2 and T11-12, at T12-L1, and at L12-L4, respectively. The genotype distributions of the SNPs were compared among these three groups and between A, B, and C, respectively. There was no statistical difference between the genotype distributions of all of 12 SNPs and location of main curve.

## 4. Discussion

Adolescent idiopathic scoliosis is a three-dimensional deformity of the spine with lateral curvature combined with vertebral rotation, which can directly affect the patient's physical and mental health [20, 21]. However, the etiology of adolescent idiopathic scoliosis is still unclear. Currently, AIS is thought to be multifactorial disorder; differing degrees of interaction between multiple factors depend on linear and summation causality [22, 23]. Although AIS has been linked to multifactorial causes, genetic factors are considered the most important cause [24]. Zhao et al. have reported that two SNPs of CALM 1 are associated with susceptibility to adolescent idiopathic scoliosis. The susceptibility of Peking Union Medical College (PUMC) classification type II (double curve) AIS and lumbar curve might be related to CALM1 rs12885713 site polymorphism, while rs5871 site polymorphism might be a risk indicator for thoracic curve cases [19]. These results give us more confidence to further explore more SNPs in CALM1 which may be associated with AIS. Therefore, we screened another 12 SNPs in CALM1 gene from 146 patients with AIS and 146 control cases and conducted an association analysis between genotype polymorphisms of CALM1 and the different clinical phenotypes of AIS. Finally, three SNPs were identified to have possible association with predisposition of AIS.

SNPs can be divided into two categories: coding SNP, located at genomic coding region, and noncoding SNP, at noncoding region. Noncoding SNPs are distributed at intron, 5′ untranslating region (UTR), 3′-UTR, and other noncoding regions [25]. SNPs located at 5′-UTR may participate in the process of transcription and translation of the regulatory gene [26], SNPs located at 3′-UTR could affect mRNA stability and translation procession [27], and SNPs located at intron may influence the process of pre-mRNA splicing [28]. SNPs located at these regions may induce quantitative and qualitative alterations in protein expression and thus correlates with some diseases, which are often referred to as functional polymorphism [9]. Rs2300496 and rs2300500 are located at the intron 1 region in CALM1 gene, and rs3213718 is located at intron 3 region. Currently, no study about the specific function of these three SNPs has been carried out.

Association between genetic marker and disease may result from the following two cases: (1) genetic marker correlates with the pathogenesis and pathology of the disease; (2) genetic marker has strong linkage disequilibrium with pathogenic sites [29, 30]. In this study, we found that the allele frequency distributions of rs2300496, rs2300500, and rs3213718 were statistically different between case group and control group (*P* = 0.0079, 0.0079, and 0.0257, resp.); the genotype distributions of rs2300496, rs2300500, and rs3213718 were statically different between case group and control group (*P* = 0.0196, 0.0196, and, 0.0378 resp.).

Thus we speculated that these three SNPs may not correlate with the pathology of AIS but possess strong linkage disequilibrium with certain pathogenic gene, and on the other hand, we deduced that base change of these three SNPs might affect the expression of CALM1 gene which leads to the increased expression of CaM, resulting in the occurrence of scoliosis. Further analysis indicated that (1) C allele of rs2300496, G allele of rs2300500, and C allele of rs3213718 correlated with increased susceptibility to adolescent idiopathic scoliosis and (2) C/C allele of rs2300496 loci (SNP01), G/G of rs2300500 loci (SNP02), and allele C/C of rs3213718 loci (SNP05) were related to AIS predisposition. In the unconditional logistic regression analysis, after adjustment for age and gender, these three SNPs has significant odds ratio. These all supported that CALM1 gene was likely to be a susceptibility gene associated with the etiology of AIS. Compared to the genetic susceptible factors, SNP sites would not produce an effect on the occurrence and development of diseases independently, they functioned together and intrinsically correlated with each other, haplotype was a reflection of genetic linkage, and, therefore, study of the haplotype could reveal the association between multiple SNPs loci and disease susceptibility [13–15]. Based on the haplotype analysis of the SNPs, frequencies were higher than 10%, and its haplotype distributions were different between case group and control group, and we got 1 positive haplotype: SNP8C-SNP01A-SNPl3C-SNPl4C-SNP02C-SNP3T-SNP4G-SNP05T-SNP011C; this again showed that CALM1 gene was possible to be a susceptibility gene of AIS.

In the study of association between CALM1 polymorphism and location of primary curve for patients with AIS, the genotype distributions of all of 12 SNPs were not found to be different among group A (thoracic curve), group B (thoracolumbar curve), and group C (lumbar curve) (*P* > 0.05). We also confirmed that there was not statistical difference between 12 SNPs of CALM1 gene and the main lateral Cobb angle.

As mentioned above, CaM was not only related to the development of AIS, but also associated with the severity and progression of AIS. Mototani et al. found that −16C > T loci (rsl2885713) were localized within a core promoter region of CALM1, and the nonsusceptible −16C allele showed increased expression levels of CaM in vivo relative to the susceptible −16T allele [31]. However, we failed to design compatible primers for rsl2885713, and we did not test it in the current study.

As a multifractorial inheritance disease, one or more genotypes change will lead to different phenotypes. Age, gender, and environmental factors also contribute to the diversity of gene phenotypes. Certain nongenetic risk factors for AIS, such as osteopenia and late menarche, have been reported. However, these data were not collected in the present study, which limited our ability to evaluate gene-environment interaction [2]. In conclusion, candidate genes which correlated with abnormalities will not be confirmed if the ranges of the gene phenotypes are not controlled.

In addition, when conducting association analysis, difference in allele frequency from systematic errors between case group and control group may result in false-positive results. Those results due to the mismatch in race and region, gender, and age structure between case group and control group only reflect the variation in evolution history, population migration, gender, and age structure between different populations [32–35]. This hierarchical phenomena existing in the population will cause a poor repeatability in the genetic study of complex diseases, leading to false-positive results. Although we screen the participants of AIS group and control group with strict inclusion criteria in this study, we still cannot ensure that there is no such interference of hierarchical factors. Consequently, more samples or nuclear families are badly needed to confirm our conclusions.

## 5. Conclusion

Despite many controversies and unanswered questions about AIS, the most difficult one is the aetiopathogenesis of AIS. We do not know if AIS is the result of one entity or more factors. The identification of aetiopathogenetic factors will enable improved prediction of progression and could aid in the development of more specific treatments. In our study, we found that genetic variants of CALM1 gene are associated with AIS and may play an important role in the development of AIS. Although it is too early to draw a conclusion, it was suggested that different clinical phenotypes of AIS might be related to different SNP loci of CAML1.

## Figures and Tables

**Table 1:** Age distribution and sex proportion between case group and control group.

Item	Case	Control group	*P* value
Female/male	121/25	121/25	>0.05
Age at diagnosis (years)	15.18 ± 2.59	N.A.	N.A.
Age range (years)	9–31	10–30	>0.05
Mean MCA^a^ ± SD^b^ (°)	52.24 ± 13.11	N.A.	N.A.
MCA range (°)	31–130	N.A.	N.A.

^a^The maximum Cobb angle (MCA).

^b^Standard deviation (SD).

**Table 2:** Primer sets and Tm for the SNaPshot assay.

	rs number	Strand	Primer sequence	Tm (°C)
SNP01	rs2300496	Forward primer	TAGATTGCCAGATGCGACT	60
		Reverse primer	AAACGCAGTCGGTGATGT	
		Genotyping primer	CACCTFGGTGTCGATACCTAGCCTCGCTGGGTGTGTTTTCCACAG	
SNP02	rs2300500	Forward primer	CTTCTGAATCATAGGATAGTTTGACC	60
		Reverse primer	TAAGTASAAAAAATAAAGATACAGCAACTG	
		Genotyping primer	CGACTGTAGGTGCGTAACTCAGGGCATTAGCCATTGTAATGGTAG	
SNP03	rs2268433	Forward primer	AGGTTTCAAAGGATTTTAAGATTGA	60
		Reverse primer	GTTATCATTCAGTCCACGACATG	
		Genotyping primer	AGCGATCTGCGAGACCGTATCAATATTAAAAGTATAACTGGAAAA	
SNP04	rs2300502	Forward primer	TCCTCTGCAGCAAACACAT	60
		Reverse primer	TGAAATTAAATAGCCATAGAAAGTGC	
		Genotyping primer	AGGGTCTCTACGCTGACGATTTTTATGGTAACTTCTAAGAGTGCT	
SNP05	rs3231718	Forward primer	TTTCTTCCCTCGGAGTGG	60
		Reverse primer	TATTTAATTATACAAAGGAAACTGCACA	
		Genotyping primer	GTGATTCTGTACGTGTCGCCGCGGAGTGGTGTATGCTATTTTTCT	
SNP06	rs3814843	Forward primer	GTCAACATCTCCTCTACCAA	60
		Reverse primer	GGGGTGGGTCAGAGCAGT	
		Genotyping primer	AGAGCGAGTGACGCATACTACACCCAGCCCAAGGACCAGTAGCAG	
SNP07	rs1058903	Forward primer	ATAAGAAACTGCTGAGTACTTGGC	60
		Reverse primer	AGTACATCAAAAGCCCATC	
		Genotyping primer	GGCTATGATTCGCAATGCTTACATCTGTTTGGAATAGGTGTTGAG	
SNP08	rs3814847	Forward primer	TGCCTGTACATTTTTCCTTTTT	60
		Reverse primer	ATGCTGTGCTAGAAAGGGC	
		Genotyping primer	CGTGCCGCTCGTGATAGAATCATATTCCTGCAGACTTTGTTGAAA	
SNP09	rs3814845	Forward primer	TTTATAATTACACACAAAATTCACTCCA	60
		Reverse primer	ATTTATGAAATAACTTAGCCKTCCTCC	
		Genotyping primer	GGATGGCGTTCCGTCCTATTGAAAAAAGCAAAGTCAAGTCAACTT	
SNP10	rs3179089	Forward primer	TTAGTGGTCCTGCAGCGTA	60
		Reverse primer	AAAACTGGAACTCAGCATTCC	
		Genotyping primer	AGATAGAGTCGATGCCAGCTACAGGATTCTTGTATGGCCAGGATC	
SNP11	rs2300497	Forward primer	GAAAGATCCGTTTATAATTTGTAAAAAC	60
		Reverse primer	CCAATTACAAAATAAGTCCTAATAGAAAG	
		Genotyping primer	GCGGTAGGTTCCCGACATATTACTGTTAAAGTTTTCACTTAAGAG	
SNP12	rs2300499	Forward primer	AACAGTTCTTATGTCATTCTTAAGGTAG	60
		Reverse primer	AAGTGTTTCAAAACTGCAAAAAT	
		Genotyping primer	ACGCACGTCCACGGTGATTTCTCTCCAGCAAATTTAGTAGATACT	

**Table 3:** Association analysis of genotype distribution of 12 SNPs.

SNP loci	Major/minor allele	Location	Case group			Control group			*P* value (*P*_adjust_^e^)		OR^c^ (95% CI)^d^
			1/1	1/2	2/2	1/1	1/2	2/2	Genotype^a^	Allele^b^	
SNP01	C/A	Intron 1	1 (1%)	35 (24%)	110 (75%)	7 (5%)	47 (32%)	92 (63%)	**0.0196**	**0.0079**	**0.5495 (0.3519**–**0.8579)**
SNP02	G/C	Intron 1	1 (1%)	35 (24%)	110 (75%)	7 (5%)	47 (32%)	92 (63%)	**0.0196**	**0.0079**	**0.5495 (0.3519**–**0.8579)**
SNP03	T/C	Intron 1	11 (8%)	59 (41%)	75 (52%)	12 (8%)	50 (34%)	84 (58%)	0.5240	0.4800	1.1417 (0.7903–1.6494)
SNP04	G/A	Intron 3	6 (4%)	59 (40%)	81 (55%)	6 (4%)	51 (35%)	89 (61%)	0.6193	0.4311	1.1678 (0.7936–1.7184)
SNP05	C/T	Intron 3	103 (71%)	42 (29%)	1 (1%)	88 (60%)	51 (35%)	7 (5%)	**0.0378**	**0.0257**	**1.6139 (1.0574**–**2.4634)**
SNP06	A/C	Exon 6	122 (84%)	23 (16%)	1 (1%)	122 (84%)	24 (16%)	0 (0%)	0.6001	0.8814	1.0456 (0.5824–1.8771)
SNP07	C/T	Exon 6	107 (73%)	35 (24%)	4 (3%)	103 (71%)	42 (29%)	1 (1%)	0.2847	0.9075	1.0274 (0.6514–1.6203)
SNP 08	C/G	5′near gene	80 (55%)	58 (40%)	8 (5%)	85 (58%)	52 (36%)	9 (6%)	0.7643	0.7010	0.9289 (0.6375–1.3535)
SNP09	C/G	Exon 6	100 (68%)	42 (29%)	4 (3%)	102 (70%)	41 (28%)	3 (2%)	0.9164	0.7387	0.9285 (0.6004–1.4359)
SNP10	C/G	Exon 6	102 (70%)	41 (28%)	3 (2%)	93 (64%)	45 (31%)	8 (5%)	0.2376	0.1357	1.3765 (0.9038–2.0966)
SNP11	C/T	Intron 1	80 (56%)	58 (40%)	6 (4%)	89 (61%)	51 (35%)	6 (4%)	0.6328	0.4342	0.8568 (0.5815–1.2624)
SNP12	C/G	Intron 1	23 (16%)	71 (49%)	51 (35%)	34 (23%)	67 (46%)	45 (31%)	0.2711	0.1517	0.7865 (0.5662–1.0925)

^a^ *P* values were calculated using the Cochran-Armitage trend test.

^b^ *P* values were calculated using the *χ* ^2^ test.

^c^Allelic odds ratio.

^d^Confidence interval (CI).

^e^ *P* values were adjusted using the Bonferroni method for multiple tests.

The bold font means *P* < 0.05.

**Table 4:** Haplotype analysis of CALM1 gene.

Haplotype	Frequencies		*χ* ^2^	*P*
	Case group	Control group		
CCCGGTGCC	174 (59.6)	156 (53.4)	2.256	0.1331
GCTCGCACG	50 (17.1)	47 (16.1)	0.111	0.7387
**CACCCTGTC**	**37 (12.7)**	**60 (20.5)**	**6.54**	**0.0105**
GCTCGCACC	21 (7.2)	16 (5.5)	0.721	0.3957
CCCCGCGTC	7 (2.4)	4 (1.4)	0.834	0.3612
GCCCGCGCC	2 (0.7)	7 (2.4)	2.2821	0.093

The SNP sequence of haplotype is SNP8-SNP01-SNPl3-SNPl4-SNP02-SNP3-SNP4-SNP05-SNP011.

The bold font means *P* < 0.05.

**Table 5:** Association analysis between genotype distributions of the SNPs and sex in case group.

SNP loci	Male			Female			*P*
	1/1	1/2	2/2	1/1	1/2	2/2	
SNP01	0	7	17	1	28	93	0.7418
SNP02	0	7	17	1	28	93	0.7418
SNP03	0	9	14	11	50	61	0.2819
SNP04	0	9	15	6	50	66	0.4734
SNP05	17	7	0	86	35	1	0.9054
SNP06	21	3	0	101	20	1	0.8013
SNP07	21	3	0	86	32	4	0.2064
SNP08	15	9	0	65	49	8	0.4276
SNP09	19	5	0	81	37	4	0.3881
SNP10	16	7	1	86	34	2	0.7127
SNP11	14	9	0	66	49	6	0.5250
SNP12	4	8	11	19	63	40	0.3000

## References

[1] Mehta MH (1992). The conservative management of Juvenile idiopathic scoliosis. *Acta Orthopaedica Belgica*.

[2] Gao W, Peng Y, Liang G (2013). Association between common variants near *LBX1* and adolescent idiopathic scoliosis replicated in the Chinese Han population. *PLoS ONE*.

[3] Weinstein SL (1999). Natural history. *Spine*.

[4] Pollak L, Shlamkovic N, Minewicz A, Mirovsky Y (2013). Otolith dysfunction as a possible cause for the development of idiopathic scoliosis. *Journal of Pediatric Orthopedics*.

[5] Azeddine B, Letellier K, Wang DS, Moldovan F, Moreau A (2007). Molecular determinants of melatonin signaling dysfunction in adolescent idiopathic scoliosis. *Clinical Orthopaedics and Related Research*.

[6] Moreau A, Wang DS, Forget S (2004). Melatonin signaling dysfunction in adolescent idiopathic scoliosis. *Spine*.

[7] Inoue M, Minami S, Nakata Y (2002). Association between estrogen receptor gene polymorphisms and curve severity of idiopathic scoliosis. *Spine*.

[8] Wu J, Qiu Y, Zhang L, Sun Q, Qiu X, He Y (2006). Association of estrogen receptor gene polymorphisms with susceptibility to adolescent idiopathic scoliosis. *Spine*.

[9] Zhang H-Q, Lu S-J, Tang M-X (2009). Association of estrogen receptor *β* gene polymorphisms with susceptibility to adolescent idiopathic scoliosis. *Spine*.

[10] Chen Z, Tang NLS, Cao X (2009). Promoter polymorphism of matrilin-1 gene predisposes to adolescent idiopathic scoliosis in a Chinese population. *European Journal of Human Genetics*.

[11] Qiu XS, Tang NLS, Yeung HY (2007). Melatonin receptor 1B (MTNR1B) gene polymorphism is associated with the occurrence of adolescent idiopathic scoliosis. *Spine*.

[12] Wang H, Wu Z, Zhuang Q (2008). Association study of tryptophan hydroxylase 1 and arylalkylamine N-acetyltransferase polymorphisms with adolescent idiopathic scoliosis in Han Chinese. *Spine*.

[13] Aulisa L, Papaleo P, Pola E (2007). Association between IL-6 and MMP-3 gene polymorphisms and adolescent idiopathic scoliosis: a case-control study. *Spine*.

[14] Carafoli E (1987). Intracellular calcium homeostasis. *Annual Review of Biochemistry*.

[15] Hanley RM, Shenolikar S, Pollack J, Steplock D, Weinman EJ (1990). Identification of calcium-calmodulin multifunctional protein kinase II in rabbit kidney. *Kidney International*.

[16] Cantaro S, Calo L, Vianello A, Favaro S, Rossi GP, Borsatti A (1985). Platelet calmodulin concentration and phospholipase A2 activity in essential hypertension. *Regulatory Peptides. Supplement*.

[17] Kindsfater K, Lowe T, Lawellin D, Weinstein D, Akmakjian J (1994). Levels of platelet calmodulin for the prediction of progression and severity of adolescent idiopathic scoliosis. *The Journal of Bone and Joint Surgery. American*.

[18] Lowe T, Lawellin D, Smith D (2002). Platelet calmodulin levels in adolescent idiopathic scoliosis: do the levels correlate with curve progression and severity?. *Spine*.

[19] Zhao D, Qiu G-X, Wang Y-P (2009). Association of calmodulin1 gene polymorphisms with susceptibility to adolescent idiopathic scoliosis. *Orthopaedic Surgery*.

[20] Zhi-Wei W, Wei-Jun W, Ming-Hui S (2013). Characteristics of the pelvic axial rotation in adolescent idiopathic scoliosis: a comparison between major thoracic curve and major thoracolumbar/lumbar curve. *The Spine Journal*.

[21] Lee JZ, Lam DJ, Lim KB (2014). Late presentation in adolescent idiopathic scoliosis: who, why, and how often?. *Journal of Pediatric Orthopedics B*.

[22] Kou I, Takahashi Y, Johnson TA (2013). Genetic variants in *GPR126* are associated with adolescent idiopathic scoliosis. *Nature Genetics*.

[23] Ryzhkov II, Borzilov EE, Churnosov MIP, Ataman AVP, Dedkov AA, Polonikov AVP (2013). Transforming growth factor beta 1 is a novel susceptibility gene for adolescent idiopathic scoliosis. *Spine*.

[24] Ogura Y, Takahashi Y, Kou I (2013). A replication study for association of 5 single nucleotide polymorphisms with curve progression of adolescent idiopathic scoliosis in Japanese patients. *Spine*.

[25] Liu Y, Lee YF, Ng MK (2011). SNP and gene networks construction and analysis from classification of copy number variations data. *BMC Bioinformatics*.

[26] Radhakrishnan AK, Raj VL, Tan LK, Liam CK (2013). Single nucleotide polymorphism in the promoter of the human interleukin-13 gene is associated with asthma in Malaysian adults. *BioMed Research International*.

[27] Jiang L, Gan L, Chen J, Wang M (2013). Genetic analysis of clinical VZV isolates collected in China reveals a more homologous profile. *BioMed Research International*.

[28] Mosor M, Ziolkowska-Suchanek I, Nowicka K, Dzikiewicz-Krawczyk A, Januszkiewicz-Lewandowska D, Nowak J (2013). Germline variants in MRE11/RAD50/NBN complex genes in childhood leukemia. *BMC Cancer*.

[29] Cardon LR, Bell JI (2001). Association study designs for complex diseases. *Nature Reviews Genetics*.

[30] König IR, Schäfer H, Müller H-H, Ziegler A (2001). Optimized group sequential study designs for tests of genetic linkage and association in complex diseases. *The American Journal of Human Genetics*.

[31] Mototani H, Mabuchi A, Saito S (2005). A functional single nucleotide polymorphism in the core promoter region of CALM1 is associated with hip osteoarthritis in Japanese. *Human Molecular Genetics*.

[32] Phillips TJ, Hen R, Crabbe JC (1999). Complications associated with genetic background effects in research using knockout mice. *Psychopharmacology*.

[33] Ackert-Bicknell CL, Shockley KR, Horton LG, Lecka-Czernik B, Churchill GA, Rosen CJ (2009). Strain-specific effects of rosiglitazone on bone mass, body composition, and serum insulin-like growth factor-I. *Endocrinology*.

[34] Gallagher EM, O’Shea DM, Fitzpatrick P (2008). Recurrence of urothelial carcinoma of the bladder: a role for insulin-like growth factor-II loss of imprinting and cytoplasmic E-cadherin immunolocalization. *Clinical Cancer Research*.

[35] Harris TG, Burk RD, Yu H (2008). Insulin-like growth factor axis and oncogenic human papillomavirus natural history. *Cancer Epidemiology, Biomarkers & Prevention*.

